# Neutrophil-to-eosinophil ratio as a biomarker for clinical outcomes in advanced stage melanoma patients treated with anti-PD-1 therapy

**DOI:** 10.1111/pcmr.13109

**Published:** 2023-07-07

**Authors:** Vincent Pozorski, Yeonhee Park, Yusuf Mohamoud, Dahlia Tesfamichael, Hamid Emamekhoo, Alexander Birbrair, Mark R. Albertini, Vincent T. Ma

**Affiliations:** 1 University of Wisconsin School of Medicine & Public Health, Madison, Wisconsin, USA; 2 Department of Biostatistics and Medical Informatics, University of Wisconsin-Madison, Madison, Wisconsin, USA; 3 Carbone Cancer Center, University of Wisconsin-Madison, Madison, Wisconsin, USA; 4 Department of Internal Medicine, Division of Hematology, Medical Oncology, and Palliative Care, University of Wisconsin-Madison, Madison, Wisconsin, USA; 5 Department of Dermatology, University of Wisconsin-Madison, Madison, Wisconsin, USA; 6 William S. Middleton Memorial Veterans Hospital, Madison, Wisonsin, USA

**Keywords:** biomarker, immune checkpoint inhibitors, immunotherapy, melanoma, metastatic melanoma, neutrophil-to-eosinophil ratio, neutrophil-to-lymphocyte ratio

## Abstract

Neutrophil-to-lymphocyte ratios (NLR) and eosinophil counts are associated with improved survival in melanoma patients treated with immune checkpoint inhibitors, but no study has investigated neutrophil-to-eosinophil ratios (NER) as a predictive indicator in this population. In this retrospective study evaluating anti-PD-1 treated patients with advanced melanoma, progression-free survival (PFS), overall survival (OS), objective response rates (ORR), and risk of high-grade (grade ≥3) immune-related adverse events (irAEs) were compared between groups defined by median pretreatment NLR and NER as well as median NLR and NER at 1-month post-treatment. Lower baseline NLR and NER were associated with improved OS [HR: 0.504, 95% CI: 0.328–0.773, *p* = .002 and HR: 0.442, 95% CI: 0.288–0.681, *p* < .001, respectively] on univariate testing. After accounting for multiple covariates, our multivariate analysis found that lower pretreatment NER was associated with better ORR (by irRECIST) (OR: 2.199, 95% CI: 1.071–4.582, *p* = .033) and improved OS (HR: 0.480, 95% CI: 0.296–0.777, *p* = .003). Baseline NLR, 1-month NLR, and 1-month NER were not associated with ORR, PFS, or OS outcomes; but 1-month NER correlated with lower risk of grade ≥3 irAEs (OR: 0.392, 95% CI: 0.165–0.895, *p* = .029). Our findings suggest baseline NER merits additional investigation as a novel prognostic marker for advanced melanoma patients receiving anti-PD-1-based regimens.

## 1 | INTRODUCTION

Immune checkpoint inhibitor (ICI)-based therapies, specifically anti-cytotoxic T lymphocyte-associated antigen-4 (anti-CTLA-4) and anti-programmed death-1 (anti-PD-1) antibodies, have transformed our understanding and management of melanoma in recent years. Compared to previously dismal outcomes with a median survival of <1 year, studies of anti-PD-1 monotherapy and combination anti-CTLA-4/PD-1 therapy in advanced melanoma have reported 6.5-year-overall survival (OS) rates of 42% and 49%, respectively (Wolchok et al., 2022). At this time, there is no validated biomarker to guide clinicians in determining which advanced melanoma patients are more likely to benefit from ICI treatment.

Numerous potential biomarkers have been evaluated for their prognostic and predictive value in identifying outcomes in patients with advanced melanoma treated with ICIs. The utility of most has been limited by costly and highly specialized testing, complicated procedures, and significant variability. A growing body of evidence suggests that simple peripheral blood markers may be a dependable and economical source of insight when evaluating which patients are more likely to benefit from ICI therapy (Li et al., 2022).

Considering the intent of improving T cell-mediated destruction of tumor cells via anti-CTLA-4/PD-1 antibodies, it is plausible that increased absolute lymphocyte counts and the presence of tumor infiltrative lymphocytes are associated with improved ICI response (Martens et al., 2016; Tumeh et al., 2014). Knowing the effect of specific neutrophil phenotypes on suppressing T-cell proliferation and promoting T cell apoptosis, studies have reported increased neutrophil counts being associated with worse ICI treatment response and outcomes (Cohen et al., 2020; de Kleijn et al., 2013; Ferrucci et al., 2016). Subsequent investigation has been focused on evaluating pretreatment neutrophil-to-lymphocyte ratio (NLR) as a potential biomarker for treatment outcomes in advanced melanoma and other solid tumors.

The utility of NLR as a prognostic tool has been debated. A recent meta-analysis of 18 studies involving over 2000 patients with melanoma receiving immunotherapy demonstrated worse OS and progression-free survival (PFS) in individuals with higher pretreatment NLR (Li et al., 2022). To date, there have not been randomized controlled trials or prospective studies comparing outcomes based on baseline NLR and other peripheral blood markers between patients receiving ICI versus alternative treatments. There is ongoing debate that NLR may provide more prognostic information regarding patient outcomes rather than being predictive of therapeutic response to ICIs.

Pretreatment neutrophil-to-eosinophil ratio (NER) was recently identified to correlate with improved outcomes in patients with metastatic renal cell carcinoma treated with nivolumab plus ipilimumab (Tucker et al., 2021). Compared to patients with NER greater than the median (>mNER), patients with NER less than the group median (≤mNER) had a significantly greater objective response rate (ORR; ≤mNER 40% versus >mNER 21.8%) and PFS (≤mNER 8.6 months versus >mNER 3.2 months; Tucker et al., 2021). Median OS was not reached in the ≤mNER group, which significantly differed from OS in the >mNER group (27.3 months; Tucker et al., 2021). The authors suggest the role of eosinophils in the immune-mediated antitumor response via their ability to recruit T cells to the tumor microenvironment and the potential for direct killing of tumor cells (Carretero et al., 2015; Rosner et al., 2018; Tucker et al., 2021). Prior studies have reported an association between greater eosinophil levels and improved survival outcomes in anti-CTLA-4-treated melanoma patients compared to those receiving chemotherapy, suggesting a potential role for baseline eosinophil count as a predictive biomarker (Ferrucci et al., 2017). Baseline eosinophil counts have also been found to be positively associated with certain immune-related adverse events (irAEs) in ICI-treated melanoma patients (Nakamura et al., 2019).

In this retrospective study, we attempt to elucidate the utility of pretreatment NER in advanced melanoma patients receiving anti-PD-1-based therapy as a biomarker of ORR, PFS, OS, and risk of high-grade (grade ≥3) irAEs. We also evaluated pretreatment NLR to further validate its ongoing use as a biomarker. NER and NLR at one-month follow-up visits were evaluated to determine if they correlate with clinical outcomes at an early timepoint after treatment initiation.

## 2 | METHODS

One hundred eighty-three patients with unresectable stage III and IV melanoma treated with anti-PD-1 monotherapy (nivolumab or pembrolizumab) or combination ipilimumab/nivolumab (I/N) at the University of Wisconsin Carbone Cancer Center between 2011 and 2022 were eligible for the retrospective analysis. Only patients with available complete blood counts with differentials were included in analyses. Patients with primary uveal melanoma were excluded.

All study procedures were approved by the University of Wisconsin Institutional Review Board. A retrospective review of electronic medical records was completed and verified for each patient by two separate independent evaluators. Demographic data included date of birth, date of death, if applicable, and gender. Treatment information included history of prior adjuvant treatment, number of prior lines of therapy, date of initiation and cessation of anti-PD-1-based regimen, treatment-related toxicities (per CTCAE v5.0), objective response rate, and date of disease progression. Physiologic data gathered included BRAF mutation status, primary melanoma type, presence of liver or brain metastasis, preexisting autoimmune disease, ECOG performance status, presence of recent infection assessed by antibiotic use within 30 days of treatment initiation, pretreatment lactate dehydrogenase (LDH) levels, and complete blood count with differential prior to treatment initiation (baseline) and at one-month post-treatment.

OS was defined as the time between treatment initiation and death or censor date (date of the last clinical note available), while PFS was defined as the time from treatment initiation until radiographic progression (per RECIST v1.1 criteria). Objective response rate was determined by retrospective review of radiographic imaging using RECIST v1.1 (Eisenhauer et al., 2009) and irRECIST criteria. The irRECIST criteria was also used to evaluate ORR (irORR) given the potential for pseudoprogression on initial follow up scans (Manitz et al., 2022). The irORR was utilized throughout all univariate and multivariate analyses other than PFS, which utilized RECIST v1.1. Rates of irAEs were evaluated per CTCAE v5.0 with a focus on high-grade toxicities (grade ≥3).

The NER and NLR were calculated by dividing the absolute neutrophil count (ANC) by the absolute eosinophil count (AEC) and absolute lymphocyte count (ALC), respectively. For patients with an AEC of zero, AEC was adjusted to 0.01 × 10/μL (the lowest reportable level) to prevent an infinite result when calculating NER, as reported previously (Tucker et al., 2021). Similarly, NER and NLR at one-month follow up were calculated using the respective measures at those time points.

Patients were divided into groups by median NLR and NER prior to ICI initiation. Demographic and patient characteristics were compared between subgroups based on Chi-squared test or Fisher’s exact test. Univariate and multivariate Cox proportional hazard models were used to investigate the association with OS and PFS, whereas logistic regression models were used to evaluate group differences in ORR by RECIST v1.1, irORR, and risk of high-grade irAEs. Multivariate analyses accounted for the following covariates: Anti-PD-1 therapy type, prior adjuvant treatment, primary melanoma type, presence of brain or liver metastasis, presence of autoimmune disease or recent infection prior to treatment, and pretreatment LDH. Kaplan–Meier estimates of overall and PFS with 95% pointwise confidence interval bands are provided. The 95% pointwise confidence interval band of the survival curves were obtained using Greenwood’s method. Log-rank tests were used to compare the difference in survival curves between groups. A biostatistician (Y.P.) performed all statistical analyses using R version 4.2.1 software (R Core Team, 2022). To account for multiple comparisons when performing univariate analyses, statistical significance was adjusted utilizing the Bonferroni method with an initial *p*-value of 0.05 (i.e., 0.5/Number of comparisons). For all other analyses, statistical significance was defined with a p-value <0.05.

**FIGURE 1:**
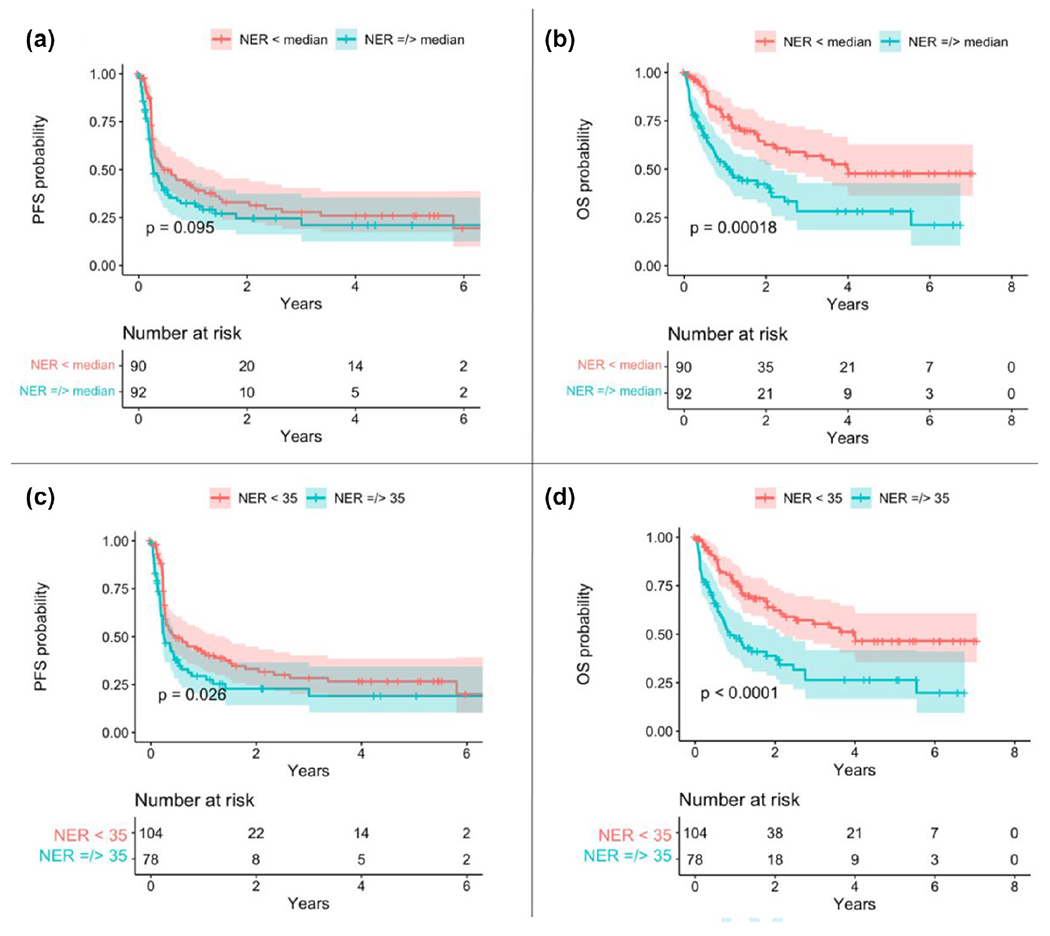
Kaplan–Meier curves of PFS and OS based on pre-treatment NER. Kaplan–Meier curves comparing PFS and OS between patients based on baseline NER. (a) PFS difference between patients with <mNER and ≥ mNER. (b) OS difference between <mNER and ≥ mNER. (c) PFS difference between patients with NER below and above the explicit cutoff of 35 at baseline. (d) OS difference between patients with NER below and above the cutoff of 35.

**FIGURE 2:**
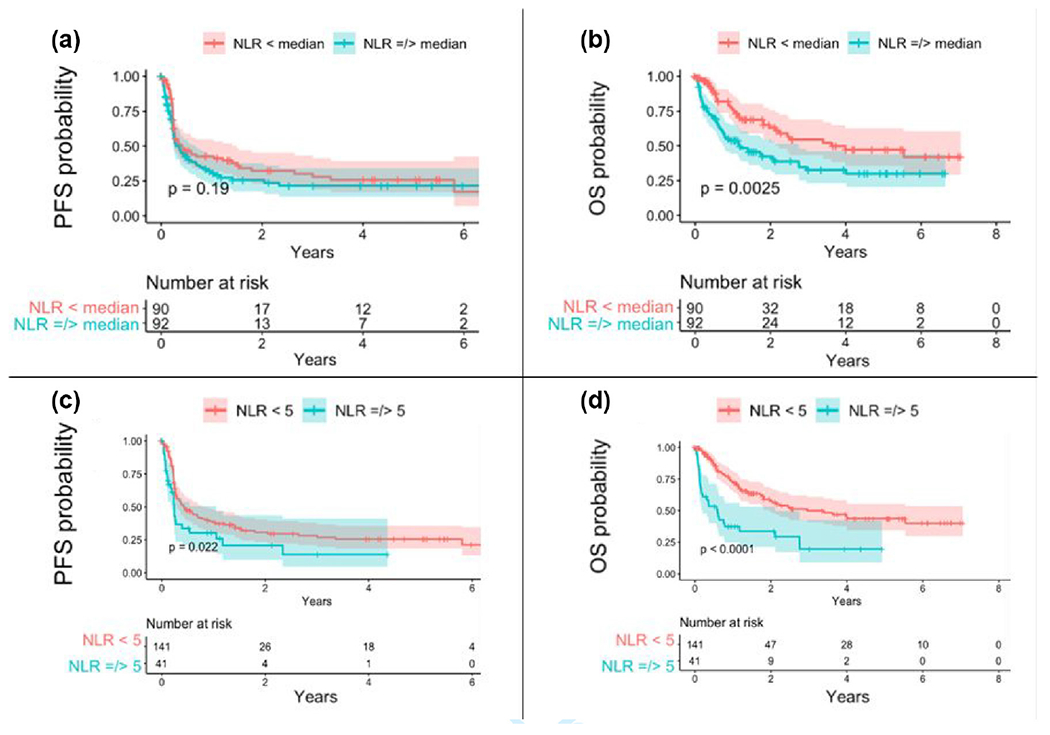
Kaplan–Meier curves of PFS and OS based on pre-treatment NLR. Kaplan–Meier curves comparing PFS and OS between patients based on baseline NLR. (a) PFS difference between patients with <mNLR and ≥ mNLR. (b) OS difference between <mNLR and ≥ mNLR. (c) PFS difference between patients with NLR below and above the explicit cutoff of 5 at baseline. (d) OS difference between patients with NLR below and above the cutoff of 5.

Following the initial analyses, receiver operating characteristic (ROC) curves were generated to evaluate the performance of the median baseline NER and NLR thresholds for predicting patient survival. The ROC curve for median NER (30.67) demonstrated an accuracy of 0.476 (95% CI: 0.257–0.702) with 27.3% sensitivity, 70% specificity, and area under the curve (AUC) of 0.514, while median NLR (3.18) had an accuracy of 0.545 (95% CI: 0.322–0.756) with 25% sensitivity, 90% specificity, and AUC = 0.575. In order to improve model performance, slightly higher explicit cutoffs were selected (NER = 35 and NLR = 5) and subsequently utilized in log-rank testing to evaluate the difference in survival curves between groups [Figures 1 & 2]. An NER cutoff of 35 improved accuracy to 0.714 (95% CI: 0.478–0.887) with 37.5% sensitivity, 92.3% specificity, and AUC = 0.649, while an NLR threshold of 5 resulted in an accuracy of 0.727 (95% CI: 0.498–0.893) with 80% sensitivity, 57.1% specificity, and AUC = 0.686.

## 3 | RESULTS

Two hundred twenty-three patients with unresectable stage III or stage IV melanoma treated with anti-PD-1 monotherapy (nivolumab or pembrolizumab) or combination I/N were initially identified. 21 patients with primary uveal melanoma were omitted and 19 subjects were excluded due to incomplete datasets. 183 participants met the inclusion criteria for analysis. Baseline demographic and patient characteristic data for the entire cohort and division by median baseline NER (mNER = 30.67) and NLR (mNLR = 3.18) are available in Table 1. For the entire cohort, the median age was 64, 61.7% were male, and 75.4% had primary cutaneous melanoma. 40.4% of patients were treated with combination I/N with the remainder receiving anti-PD-1 monotherapy. When comparing baseline characteristics between subgroups, more patients with ≥mNER had an elevated pretreatment LDH (*p* = .033) compared to those with <mNER. A greater number of subjects with ≥mNLR had brain metastasis (*p* = .044) and worse baseline ECOG performance status (*p* = .040) compared to those with <mNLR, respectively. No other significant difference in demographic or patient characteristic data was observed. Among the entire cohort, median PFS was 4.5 months (95% CI: 3.2–8.3 months) and median OS was 26.7 months (95% CI: 20.6–66.5 months). Our observed objective response rate was 36.6% (*n* = 67) per RECIST v1.1; and 45.9% (*n* = 84) per irRECIST. Development of grade ≥3 irAEs was observed in 27.9% (*n* = 51) of the cohort. Objective response rates did not differ between <mNER and ≥ mNER (*p* = .176) or between <mNLR and ≥ mNLR (*p* = .235) when using RECIST v1.1 criteria, but differed between mNER subgroups when using irRECIST (*p* = .004) [Table 2]. Rates of high-risk irAEs did not differ between baseline NER or NLR subgroups (p = 0.286 and p = 0.118, respectively) [Table 2].

Lower pretreatment NER correlated with better irORR on univariate and multivariate testing (OR: 2.664, 95% CI: 1.445–4.987, *p* = .002 and OR: 2.199, 95% CI: 1.071–4.582, *p* = .033) [Table 3, Table S1]. Pretreatment NLR did not correlate with irORR. Increased likelihood of irORR was associated with lower 1-month NLR (OR: 2.608, 95% CI: 1.413–4.885, *p* = .002) on univariate regression analysis, but was not present in the multivariable analysis [Tables S1]. Neither NLR at baseline or 1-month follow up were associated with high-grade irAEs, but 1-month NER did correlate with decreased risk of grade ≥3 irAEs (OR: 0.392, 95% CI: 0.165–0.895, *p* = .029) [Table 3, Tables S1].

Lower 1-month NLR was associated with improved PFS on univariate analysis (HR: 0.541, 95% CI: 0.376–0.779, *p* = .001) [Table 4]. Univariate analysis revealed improved PFS with lower NER when a cutoff of 35 was applied (*p* = .026) [Figure 1] as well as with an NLR cutoff of 5 (*p* = .022) [Figure 2]. No relationship was observed between PFS and NLR or NER at baseline or at 1-month in our multivariate analysis [Table 5, Table 6].

Our univariate results revealed an association between improved OS with lower baseline NLR (HR: 0.504, 95% CI: 0.328–0.773, p = 0.002), lower baseline NER (HR: 0.442, 95% CI: 0.288–0.681, *p* < 0.001), and lower 1-month NLR (HR: 0.362, 95% CI: 0.228–0.573, *p* < 0.001) [Table 4]. Univariate log-rank testing for the generation of Kaplan–Meier curves also demonstrated a significant association between lower baseline NLR and NER and improved OS (*p* = 0.0025 and *p* < 0.00018, respectively) [Figure 1, Figure 2]. Log-rank testing with a higher explicit cutoff for NLR (i.e., <5 versus ≥5) and NER (<35 versus ≥35) revealed an even greater association between lower NLR and NER and improved OS (*p* < .001) [Figure 1, Figure 2]. Only lower pretreatment NER correlated with improved OS upon multivariate testing (HR: 0.480, 95% CI: 0.296–0.777, *p* = .003) [Table 5, Table 6].

## 4 | DISCUSSION

Our study is the first to evaluate pretreatment NER as a prognostic biomarker in advanced melanoma patients receiving anti-PD-1 monotherapy or combined anti-CTLA-4/PD-1. Our univariate analysis revealed an association between improved OS with lower NER and NLR at baseline and NLR 1-month after reatment initiation. Subsequent multivariate analysis revealed only <mNER to be associated with improved OS. Multivariate testing also revealed an association between baseline NER with irORR and 1-month NER with lower risk of grade ≥3 irAEs.

With increased understanding of neutrophilic influence on the adaptive antitumor immune response in recent years, there has been a subsequent accumulation of evidence for the role of a combined measure, the neutrophil-to-lymphocyte ratio, as a prognostic indicator in melanoma patients treated with ICI therapy. Most notably, a recent meta-analysis of 18 studies performed by Li et al. demonstrated a strong relationship between higher NLR and worse OS (HR: 2.46, 95% CI: 1.77–3.43) and PFS (HR: 2.38, 95% CI: 1.95–2.89). In a recent report, (Guida et al., 2022). identified an association between both baseline NLR and change in NLR at 3–4 weeks after initiating treatment with PFS, OS, and disease control rate. The role of NLR as a prognostic biomarker is further supported by studies evaluating intratumoral immune cell populations. Schalper et al. investigated serum IL-8, a potent regulator of neutrophil chemotaxis, in patients receiving ICI and observed a positive correlation between circulating IL-8 and both peripheral neutrophil counts and tumor infiltrating neutrophilis as well as a negative association with response rates and survival outcomes (Schalper et al., 2020). In addition, more favorable ICI response has been identified in patients with higher levels absolute lymphocyte counts in circulating blood, CD8+ T-cells intratumorally and at the invasive margin, as well as intratumoral B-lymphocytes and tertiary lymphoid structures (Fridman et al., 2022; Martens et al., 2016; Tumeh et al., 2014). While no association between pretreatment NLR and outcomes was identified upon multivariate testing in the present study, it is important to note that sources vary regarding the cutoff used to define elevated NLR. Compared to our study, which used a median NLR cutoff of 3.18, many studies use a threshold of 5 which may explain why no group differences in outcomes were observed. This observation is further supported by our findings of significant differences in survival outcomes when an NLR threshold of 5 was used [Figure 2].

Although less substantiated in the literature compared to neutrophils, both absolute and relative eosinophil counts (REC) have been associated with outcomes in melanoma patients treated with ICIs (Ferrucci et al., 2017; Weide et al., 2016; Zahoor et al., 2018). Weide et al. observed greater OS in patients with REC ≥1.5% following treatment with pembrolizumab. When REC ≥1.5% was used in conjunction with other baseline markers including relative lymphocyte count ≥17.5%, LDH ≤2.5 ULN, and absence of metastasis other than soft tissue or lung, they observed a significantly greater one-year OS (83.9% vs. 14.7%) and overall response (58.3% vs. 3.3%) compared to individuals with none of these favorable characteristics (Weide et al., 2016). Following this report, REC was evaluated in patients receiving anti-CTLA-4 therapy and chemotherapy (Ferrucci et al., 2017). Patients treated with anti-CTLA-4 therapy with REC ≥1.5% were found to have improved OS and PFS, while the survival benefit was not seen in those receiving chemotherapy suggesting a potential predictive role for REC in those receiving ICI therapy (Ferrucci et al., 2017). While not previously investigated in melanoma, lower NER has correlated with improved OS, PFS, and ORR in metastatic renal cell carcinoma treated with combined immune checkpoint blockade (Tucker et al., 2021).

The exact contribution of eosinophils to the antitumor immune response is unknown, yet their role has shown variable outcomes based on cancer type with antitumoral action seen in most cancer types, including melanoma (Varricchi et al., 2017). Preclinical modeling has demonstrated its importance in recruiting CD8+ T cells to the tumor microenvironment via CCL5, CXCL9, and CDCL10 chemokine release (Carretero et al., 2015; Varricchi et al., 2017). Eosinophils are thought to amplify the Th1 immune response indirectly by promoting macrophage M1 skewing via IFN-γ and TNF-α production as well as contribute to the normalization of tumor vasculature resulting in tumor rejection (Biswas & Mantovani, 2010; Varricchi et al., 2017). Furthermore, IL-33-actived eosinophils have also demonstrated the capacity for direct tumor cell killing via cytotoxic granule release (Lucarini et al., 2017; Varricchi et al., 2017).

The results of our study were consistent with the current literature on the role of each pertinent cell line and its clinical outcomes. Concordant with the expected antitumorigenic impact of eosinophils, we found that lower pretreatment NER was largely associated with improved survival outcomes and tumor response in ICI-treated melanoma patients. Similarly, but with less statistical significance, lower pretreatment NLR also improved survival outcomes.

IrAEs can affect nearly any organ system with severe to lethal toxicities resulting in the discontinuation of ICI therapy in an estimated 4–45% of patients, which further emphasizes the importance of identifying a biomarker for clinicians (Boutros et al., 2016). Numerous factors have been found to be associated with irAEs in patients treated with ICIs, including younger age (<60 years), elevated BMI, sex, smoking status, the presence of multiple chronic medical conditions and existing autoimmunity, certain medication use, and various potential biomarkers such as specific genotypes, cytokines, autoantibodies, and blood counts (Chennamadhavuni et al., 2022). Pertinent to the present study, higher NLR has been associated with increased irAEs in patients receiving ICI therapy for advanced non-small cell lung cancer (Pavan et al., 2019), while higher baseline AEC and an increase of eosinophils by ≥3.2% at one-month follow up were both associated with greater occurrence of endocrine adverse events (Nakamura et al., 2019). In our study, no association between NLR and irAEs was identified, which is consistent with prior reports on melanoma (Nakamura et al., 2019). Similarly, baseline NER was not associated with irAEs; however, NER at 1-month follow up was associated with lower risk of high-grade irAEs, which supports further investigation of its potential as a biomarker for treatment-related toxicity as well as survival outcomes and tumor response.

There are several limitations to our study. First, it is a relatively small non-randomized study retrospective study, which limits our ability to comment on the predictive capacity of NER on ICI-specific regimens. As a retrospective study evaluating blood counts, a significant limitation is the inaccessibility of corresponding tissue samples, which prevented our ability to correlate peripheral blood markers with intratumoral immune cell activity. Future study should evaluate both peripheral and intraturmoal immune cell populations to further assess the role of eosinophils in the antitumor immune response as well as the viability of NER as a biomarker. Furthermore, the use of peripheral leukocyte counts as a biomarker may lack specificity when determing treatment response, as they are susceptible to influence from a myriad of causes, including medications, infections, physical or emotional stressors, allergic reactions, autoimmune conditions, or other comorbidities such as hematogenous malignancies and myeloproliferative disorders. The effects of which may not have been fully controlled for within our statistical modeling. In addition, the study’s sample size is relatively small, which may have limited the statistical power of the analyses and our ability to detect differences in clinical outcomes. As this was a single-center study, external validation of our findings in other larger cohorts will be needed. Although we controlled for multiple covariates in our multivariate testing, it was noted that more patients with ≥mNER had an elevated pretreatment LDH (*p* = 0.033), while a greater number of subjects with ≥mNLR had brain metastasis (*p* = 0.044) and worse functional status (*p* = 0.040) compared to those with <mNER and < mNLR, respectively, which indicates imperfect subgroup matching. A final limitation is the relatively low OS (26.7 months) and PFS (4.5 months) observed in our study compared to historic outcomes associated with anti-PD-1 based therapy, which is felt to be a result of patients with higher acuity in our cohort as they received treatment at a tertiary referral center.

## 5 | CONCLUSION

Lower pretreatment NER is associated with improved irORR and OS in patients with advanced melanoma treated with anti-PD-1-based regimens. Although pretreatment NLR was associated with OS upon univariate testing, this relationship did not persist after controlling for pertinent clinicopathologic variables. Survival outcomes may be further delineated with higher explicit NER and NLR cutoffs. While additional studies are warranted to validate NER as a predictive marker in melanoma patients treated with ICI, our findings, in combination with the literature, suggest that the utilization of both NLR and NER may provide useful information to clinicians when attempting to identify which patients are most likely to derive a therapeutic benefit. Future study of intratumoral immune cell populations is warranted to provide additional support for the role of eosinophils in the antitumor immune response.

## Supplementary Material

Supporting Information

## Figures and Tables

**TABLE 1:** Baseline demographics and patient characteristics.

	All (*N* = 183)	<mNER (*N* = 91)	≥mNER (*N* = 92)	*p*-Value	<mNLR (*N* = 91)	≥mNLR (*N* = 92)	*p*-Value
Anti-PD-1 treatment type				0.833			0.086
Ipilimumab/Nivolumab	74	38	36		43	31	
Anti-PD-1 Monotherapy (Pembrolizumab or Nivolumab)	109	53	56		48	61	

Prior adjuvant treatment							
Yes	35	18	17	0.971	19	16	0.680
No	148	73	75		72	76	

Lines of prior therapy (median = 0)							
0	124	59	65	0.689	59	65	0.649
1	43	23	20		24	19	
≥2	16	9	7		8	8	

Age (years)							
<65	95	46	49	0.943	48	47	0.823
≥65	88	45	43		43	45	

Gender							
Male	113	55	58	0.833	54	59	0.607
Female	70	36	34		37	33	

Baseline autoimmune disease							
Yes	23	13	10	0.636	15	8	0.172
No	160	78	82		76	84	

ECOG performance status							
0–1	151	80	71	0.135	81	70	0.040*
2-4	19	8	11		5	14	
Unknown	13	3	10		5	8	

Infection prior to treatment							
Yes	27	11	16	0.422	11	16	0.422
No	156	80	76		80	76	

BRAF mutation status							
V600 mutant	71	38	33	0.217	37	34	0.694
Wildtype	109	53	56		52	57	
Unknown	3	0	3		2	1	

Primary melanoma type							
Cutaneous	138	66	72	0.466	67	71	0.700
Mucosal + Unknown	45	25	20		24	21	

Pre-treatment LDH level^‡^							
Within normal limits	103	60	43	0.033*	58	45	0.122
≥Upper limit of normal	73	28	45		30	43	
Unknown	7	3	4		3	4	

Liver metastases							
Yes	57	26	31	0.556	22	35	0.062
No	126	65	61		69	57	

Brain metastases							
Yes	45	17	28	0.094	16	29	0.044*
No	138	74	64		75	63	

*Note*: Baseline demographics and patient characteristics for the entire cohort and subgroups based on median pre-treatment NER and NLR. Chi-squared and Fisher’s exact testing performed to evaluate subgroup differences.

Abbreviations: LDH, lactate dehydrogenase; mNER, median neutrophil-to-eosinophil ratio; mNLR, median neutrophil-to-lymphocyte ratio.

* Statistical significance of *p* < 0.05.

‡ Normal limits of LDH <240 U/L.

**TABLE 2:** Objective response rates (by RECIST v1.1 and irRECIST) and immune-related adverse events.

	All (*N* = 183)	<mNER (*N* = 91)	≥mNER (*N* = 92)	*p*-Value	<mNLR (*N* = 91)	≥mNLR (*N* = 92)	*p*-Value
Objective response rate (per RECIST v1.1)							
Yes (CR + PR)	67	37	30	0.176	36	31	0.235
No (SD + PD)	102	50	52		51	51	
Unknown or N/A	14	4	10		4	10	

Objective Response Rate (per irRECIST)							
Yes (CR + PR)	84	53	31	0.004*	49	35	0.091
No (SD + PD)	86	34	52		38	48	
Unknown or N/A	13	4	9		4	9	

Immune-related adverse events							
Grade <3	131	69	62	0.286	60	71	0.118
Grade ≥3	51	22	29		30	21	
Unknown	1	0	1		1	0	

Note: Comparison of grade **≥**3 immune-related adverse events and objective response rates between subgroups based on median pre-treatment NER and NLR. Fisher’s exact testing was performed to evaluate subgroup differences.

Abbreviations: CR, complete response; irRECIST, immune-related Response Evaluation Criteria In Solid Tumors; mNER, median neutrophil-to-eosinophil ratio; mNLR, median neutrophil-to-lymphocyte ratio; N/A, not available; PD, progressive disease; PR, partial response; RECIST v1.1, Response Evaluation Criteria In Solid Tumors version 1.1; SD, stable disease.

* Indicates statistical significance of *p* < 0.05.

**TABLE 3:** Multivariate analyses of irORR and grade ≥3 irAEs by mNER at baseline and 1-month follow-up.

	Objective response rate		Grade ≥3 irAEs	
	OR (95% CI)	*p*-value	OR (95% CI)	*p*-value
Anti-PD-1 Treatment Type (I/N vs. Anti-PD-1 Monotherapy)	2.025 (0.956–4.420)	0.070	7.728 (3.368–19.218)	<0.001*
Prior Adjuvant Treatment (yes vs. no)	0.413 (0.160–1.021)	0.060	0.648 (0.238–1.643)	0.376
Baseline Autoimmune Disease (yes vs. no)	1.083 (0.391–3.036)	0.877	2.557 (0.891–7.240)	0.076
Infection Prior to Treatment (yes vs. no)	0.655 (0.215–1.900)	0.441	0.438 (0.123–1.333)	0.168
Primary Melanoma Type (cutaneous vs. mucosal + unknown)	2.469 (1.064–5.979)	0.039*	1.270 (0.526–3.220)	0.603
Pre-treatment LDH level^‡^ (≥upper limit vs. normal)	0.268 (0.124–0.561)	<0.001*	0.788 (0.347–1.751)	0.561
Brain Metastases (yes vs. no)	1.306 (0.558–3.112)	0.541	0.967 (0.385–2.328)	0.941
Liver Metastases (yes vs. no)	0.844 (0.385–1.843)	0.669	0.691 (0.282–1.615)	0.403
Pre-treatment NER (<mNER vs. ≥mNER)	2.199 (1.071–4.582)	0.033*	0.783 (0.347–1.752)	0.552
NER at 1 month (<median NER 1 mo vs. ≥median NER 1 mo)	1.107 (0.523–2.314)	0.787	0.392 (0.165–0.895)	0.029*

Note: Multivariate logistic regression models for ORR and Grade ≥3 irAEs.

Abbreviations: I/N, ipilimumab/nivolumab; irAEs, grade ≥3 immune-related adverse events; LDH, lactate dehydrogenase; mNER, median neutrophil-to-eosinophil ratio; ORR, objective response rate; OR, odds ratio.

* Statistical significance of *p* < 0.05.

‡ Normal limits of LDH <240 U/L.

**TABLE 4:** Univariate analyses of PFS and OS.

	Progression-free survival		Overall survival	
	HR (95% CI)	*p*-Value	HR (95% CI)	*p*-Value
Age (<65 vs ≥65)	0.913 (0.642–1.298)	0.613	1.075 (0.711–1.625)	0.733
Gender (male vs. female)	1.121 (0.779–1.611)	0.539	1.055 (0.692–1.608)	0.804
BRAF status (mutant vs. wildtype)	1.012 (0.703–1.458)	0.949	0.854 (0.555–1.313)	0.472
Anti-PD-1 treatment type (I/N vs. Anti-PD-1 Monotherapy)	0.679 (0.467–0.987)	0.042	0.763 (0.488–1.192)	0.234
Prior adjuvant treatment (yes vs. no)	1.197 (0.781–1.835)	0.409	0.824 (0.480–1.416)	0.484
Baseline autoimmune disease (yes vs. no)	1.204 (0.739–1.963)	0.456	0.994 (0.541–1.827)	0.986
Infection prior to treatment (yes vs. no)	1.302 (0.790–2.148)	0.301	2.292 (1.392–3.772)	0.001*
Primary melanoma type (cutaneous vs. mucosal + unknown)	1.077 (0.714–1.624)	0.725	0.812 (0.505–1.304)	0.389
Pre-treatment LDH level^‡^ (≥upper limit vs. normal)	1.923 (1.330–2.778)	0.001*	3.124 (2.019–4.832)	<0.001*
Brain metastases (yes vs. no)	1.172 (0.777–1.769)	0.448	1.564 (0.991–2.468)	0.055
Liver metastases (yes vs. no)	1.618 (1.115–2.348)	0.011	2.213 (1.451–3.376)	<0.001*
Pre-treatment NLR (<mNLR vs. ≥mNLR)	0.772 (0.541–1.102)	0.154	0.504 (0.328–0.773)	0.002*
NLR at 1 month (<median NLR 1 mo vs. ≥median NLR 1 mo)	0.541 (0.376–0.779)	0.001*	0.362 (0.228–0.573)	<0.001*
Pre-treatment NER (<mNER vs. ≥mNER)	0.722 (0.505–1.031)	0.073	0.442 (0.288–0.681)	<0.001*
NER at 1 month (<median NER 1 mo vs. ≥median NER 1 mo)	0.735 (0.512–1.054)	0.094	0.578 (0.372–0.900)	0.015

Note: Univariate Cox proportional hazard regression models for PFS and OS.

Abbreviations: HR, hazard ratio; I/N, ipilimumab/nivolumab; LDH, lactate dehydrogenase; mNER, median neutrophil-to-eosinophil ratio; mNLR, median neutrophil-to-lymphocyte ratio; OS, overall survival; PFS, progression-free survival.

* Statistical significance of *p* < 0.003 (Bonferroni correction: p = 0.05/15).

‡ Normal limits of LDH <240 U/L.

**TABLE 5:** Multivariate analyses of PFS and OS by mNER at baseline and 1-month follow-up.

	Progression-free survival		Overall survival	
	HR (95% CI)	*p*-Value	HR (95% CI)	*p*-Value
Anti-PD-1 treatment type (I/N vs Anti-PD-1 Monotherapy)	0.533 (0.345–0.821)	0.004*	0.571 (0.338–0.964)	0.036*
Prior adjuvant treatment (yes vs no)	1.738 (1.077–2.806)	0.024*	1.483 (0.819–2.688)	0.194
Primary melanoma type (cutaneous vs mucosal + unknown)	0.793 (0.502–1.253)	0.320	0.490 (0.281–0.852)	0.012*
Baseline autoimmune disease (yes vs no)	1.074 (0.641–1.800)	0.787	0.926 (0.494–1.734)	0.809
Infection prior to treatment (yes vs no)	1.142 (0.640–2.037)	0.654	1.454 (0.779–2.711)	0.240
Pre-treatment LDH level^‡^ (≥upper limit vs normal)	1.734 (1.155–2.605)	0.008*	2.473 (1.535–3.984)	<0.001*
Brain Metastases (yes vs no)	0.890 (0.554–1.429)	0.629	1.114 (0.650–1.910)	0.694
Liver Metastases (yes vs no)	1.447 (0.963–2.173)	0.075	2.089 (1.306–3.342)	0.002*
Pre-treatment NER (<mNER vs ≥mNER)	0.827 (0.553–1.236)	0.355	0.480 (0.296–0.777)	0.003*
NER at 1 month (<median NER 1 mo vs ≥median NER 1 mo)	1.015 (0.667–1.545)	0.944	0.929 (0.563–1.534)	0.774

Note: Multivariate Cox proportional hazard regression models for PFS and OS.

Abbreviations: HR, hazard ratio; I/N, ipilimumab/nivolumab; LDH, lactate dehydrogenase; mNER, median neutrophil-to-eosinophil ratio; OS, overall survival; PFS, progression-free survival.

* Statistical significance of *p* = 0.05.

‡ Normal limits of LDH <240 U/L.

**TABLE 6:** Multivariate analyses of PFS and OS by mNLR at baseline and 1-month follow-up.

	Progression-free survival		Overall survival	
	HR (95% CI)	*p*-value	HR (95% CI)	*p*-value
Anti-PD-1 treatment type (I/N vs Anti-PD-1 Monotherapy)	0.538 (0.348–0.833)	0.005*	0.589 (0.354–0.980)	0.042*
Prior adjuvant treatment (yes vs no)	1.756 (1.080–2.855)	0.023*	1.319 (0.729–2.388)	0.360
Primary melanoma type (cutaneous vs mucosal + unknown)	0.805 (0.512–1.266)	0.347	0.524 (0.305–0.901)	0.019*
Baseline autoimmune disease (yes vs no)	1.061 (0.631–1.783)	0.823	1.005 (0.528–1.912)	0.988
Infection prior to treatment (yes vs no)	1.014 (0.560–1.835)	0.964	1.591 (0.865–2.927)	0.135
Pre-treatment LDH level^‡^ (≥upper limit vs normal)	1.612 (1.063–2.446)	0.025*	2.362 (1.462–3.815)	<0.001*
Brain metastases (yes vs no)	0.876 (0.542–1.414)	0.587	0.980 (0.568–1.692)	0.943
Liver metastases (yes vs no)	1.277 (0.837–1.949)	0.257	1.752 (1.090–2.814)	0.021*
Pre-treatment NLR (<mNLR vs ≥mNLR)	1.113 (0.703–1.761)	0.649	0.731 (0.429–1.246)	0.250
NLR at 1 month (<median NLR 1 mo vs ≥median NLR 1 mo)	0.685 (0.418–1.123)	0.133	0.663 (0.367–1.198)	0.173

Multivariate Cox proportional hazard regression models for PFS and OS.

Abbreviations: HR, hazard ratio; I/N, ipilimumab/nivolumab; LDH, lactate dehydrogenase; mNLR, median neutrophil-to-lymphocyte ratio; OS, overall survival; PFS, progression-free survival.

* Statistical significance of *p* < 0.05.

‡ Normal limits of LDH <240 U/L.

## Data Availability

The data that support the findings of this study are available on request from the corresponding author. The data are not publicly available due to privacy or ethical restrictions.

## Significance

There has yet to be validation of a biomarker that can routinely help guide clinicians in determining which patients with advanced-stage melanoma are more likely to benefit from anti-PD-1-based treatment. The neutrophil-to-eosinophil ratio (NER) has been demonstrated to correlate with survival outcomes in metastatic renal cell carcinoma, but has not yet been evaluated as a potential biomarker in melanoma patients. Given the potential role that both neutrophils and eosinophils play in the antitumor immune response, NER may provide useful prognostic information regarding clinical outcomes when discussing treatment options to patients who are candidates for immune checkpoint inhibitors.
