# Supplementary material for: Neutrophil-to-eosinophil ratio as a biomarker for clinical outcomes in advanced stage melanoma patients treated with anti-PD-1 therapy
Source: Pigment Cell Melanoma Res. Author manuscript; Available in PMC 2025 Feb 25. (PMC11851330; doi:10.1111/pcmr.13109)
Supplement: Supporting Information [file NIHMS2058056-supplement-Supporting_Information.docx]

Supplementary Table 1. Univariate analyses of irORR and grade ≥3 irAEs

|  | Objective Response Rate | | Grade ≥3 irAEs | |
| --- | --- | --- | --- | --- |
|  | OR (95% CI) | p-value | OR (95% CI) | p-value |
| Anti-PD-1 Treatment Type (I/N vs Anti-PD-1 Monotherapy) | 1.504 (0.826 – 2.757) | 0.183 | 5.043 (2.589 – 10.166) | <0.001* |
| Prior Adjuvant Treatment (yes vs no) | 0.780 (0.374 – 1.612) | 0.503 | 1.408 (0.644 – 2.976) | 0.378 |
| Baseline Autoimmune Disease (yes vs no) | 1.063 (0.440 – 2.590) | 0.892 | 1.500 (0.597 – 3.579) | 0.370 |
| Recent Infection Prior to Treatment (yes vs no) | 0.533 (0.212 – 1.273) | 0.164 | 0.501 (0.161 – 1.300) | 0.186 |
| Primary Melanoma Type (cutaneous vs mucosal + unknown) | 1.507 (0.761 – 3.024) | 0.242 | 0.833 (0.413 – 1.731) | 0.616 |
| Pre-treatment LDH level‡ (≥upper limit vs normal) | 0.263 (0.133 – 0.505) | <0.001* | 0.829 (0.419 – 1.611) | 0.583 |
| Brain Metastases (yes vs no) | 0.898 (0.444 – 1.809) | 0.763 | 0.990 (0.462 – 2.035) | 0.978 |
| Liver Metastases (yes vs no) | 0.647 (0.333 – 1.242) | 0.193 | 0.861 (0.420 – 1.707) | 0.674 |
| Pre-treatment NLR (<mNLR vs ≥mNLR) | 1.805 (0.987 – 3.328) | 0.056 | 1.663 (0.868 – 3.229) | 0.128 |
| NLR at 1 month (<median NLR 1 mo vs ≥median NLR 1 mo) | 2.608 (1.413 – 4.885) | 0.002* | 1.073 (0.560 – 2.060) | 0.832 |
| Pre-treatment NER (<mNER vs ≥mNER) | 2.664 (1.445 – 4.987) | 0.002* | 0.672 (0.348 – 1.284) | 0.231 |
| NER at 1 month (<median NER 1 mo vs ≥median NER 1 mo) | 2.038 (1.111 – 3.779) | 0.022 | 0.607 (0.312 – 1.167) | 0.137 |

Univariate logistic regression models for ORR and Grade ≥3 irAEs.

*Statistical significance of p <0.004 (Bonferroni correction: p = 0.05/12). ‡Normal limits of LDH <240 U/L.

Abbreviations: I/N, ipilimumab/nivolumab; irAEs, grade ≥3 immune-related adverse events; irORR, objective response rate per irRECIST criteria; LDH, lactate dehydrogenase; mNER, median neutrophil-to-eosinophil ratio; mNLR, median neutrophil-to-lymphocyte ratio; OR, odds ratio.

Supplementary Table 2. Multivariate analyses of irORR and grade ≥3 irAEs by mNLR at baseline and 1-month follow-up

|  | Objective Response Rate | | Grade ≥3 irAEs | |
| --- | --- | --- | --- | --- |
|  | OR (95% CI) | p-value | OR (95% CI) | p-value |
| Anti-PD-1 Treatment Type (I/N vs Anti-PD-1 Monotherapy) | 1.825 (0.870 – 3.915) | 0.115 | 5.839 (2.648 – 13.687) | <0.001* |
| Prior Adjuvant Treatment (yes vs no) | 0.406 (0.159 – 0.988) | 0.051 | 0.708 (0.261 – 1.795) | 0.480 |
| Baseline Autoimmune Disease (yes vs no) | 0.971 (0.349 – 2.712) | 0.955 | 2.185 (0.762 – 6.147) | 0.138 |
| Infection Prior to Treatment (yes vs no) | 0.689 (0.229 – 2.007) | 0.498 | 0.494 (0.138 – 1.527) | 0.243 |
| Primary Melanoma Type (cutaneous vs mucosal + unknown) | 2.460 (1.075 – 5.862) | 0.036* | 1.148 (0.492 – 2.806) | 0.754 |
| Pre-treatment LDH level‡ (≥upper limit vs normal) | 0.256 (0.118 – 0.536) | <0.001* | 0.900 (0.403 – 1.980) | 0.793 |
| Brain Metastases (yes vs no) | 1.384 (0.589 – 3.320) | 0.459 | 1.103 (0.448 – 2.627) | 0.827 |
| Liver Metastases (yes vs no) | 0.989 (0.449 – 2.193) | 0.978 | 0.738 (0.301 – 1.736) | 0.495 |
| Pre-treatment NLR (<mNLR vs ≥mNLR) | 1.506 (0.687 – 3.305) | 0.305 | 1.502 (0.650 – 3.521) | 0.343 |
| NLR at 1 month (<median NLR 1 mo vs ≥median NLR 1 mo) | 1.629 (0.712 – 3.741) | 0.247 | 0.713 (0.288 – 1.729) | 0.457 |

Multivariate logistic regression models for ORR and Grade ≥3 irAEs.

*Statistical significance of p <0.05. ‡Normal limits of LDH <240 U/L.

Abbreviations: I/N, ipilimumab/nivolumab; irAEs, grade ≥3 immune-related adverse events; irORR, objective response rate per irRECIST criteria; LDH, lactate dehydrogenase; mNLR, median neutrophil-to-lymphocyte ratio; OR, odds ratio.
